# General practitioners' role in cancer care: a French-Norwegian study

**DOI:** 10.1186/1756-0500-2-200

**Published:** 2009-09-29

**Authors:** Lise Demagny, Knut Holtedahl, Janine Bachimont, Tommy Thorsen, Alain Letourmy, Martine Bungener

**Affiliations:** 1 INSERM/CERMES Centre de Recherche Médecine, Sciences, Santé et Société Site CNRS, 7, rue Guy Môquet, 94801 Villejuif, France; 2 Institute of Community Medicine, University of Tromsø, 9037 Tromsø, Norway

## Abstract

**Background:**

In cancer care, a GP's work is rarely defined clearly. Our aim was to assess GPs' work with cancer patients in France and in Norway, where the roles of the GP and the organization of the system are rather different.

**Findings:**

A questionnaire with 40 closed-ended questions about GP involvement in diagnosis, treatment, follow-up and terminal care was constructed and mailed to samples of GPs. The patients had seen the doctor at least once over the past year. In France 1679 and in Norway 386 individual patient questionnaires were completed. GPs have a major role in the diagnosis of cancer, and this role varies according to cancer type. The GPs participated actively in different phases of follow-up after cancer treatment. Low response rates do not allow direct comparison between countries, but higher PSA screening rates in France seem to increase the percentage of patients diagnosed after screening rather than after a clinical suspicion. Interaction between GPs and specialists during cancer treatment and follow-up was important in both countries.

**Conclusion:**

Both in France and in Norway GPs participate actively in cancer care. Early clinical diagnosis is a challenge. More research is needed about how GPs can improve their early diagnostic work. Organisational issues may influence cancer responsibilities for the GP, and national health systems should be challenged to look at possible new roles for GPs in cancer care. Medical training, both pre- and post-graduate, should prepare doctors for collaboration between primary and secondary care, particularly important in cancer care.

## Background

In cancer care, diagnosis [1-3], follow-up [4,5] and palliative care fall within the responsibility of the GP. Despite this, the work of the GP in this area is often considered as residual compared to that of specialists. In France and Norway, patients are free to choose their doctor, but in Norway the gatekeeper role of the GP is more pronounced and the circuit of medical professionals and health care institutions is more predetermined, depending on the patient's problem. We studied the diagnostic and therapeutic responsibility sphere of the GPs in the two countries with regard to cancer.

## Methods

### Design and setting

The questionnaire [see Additional file 1] and the protocol used were established jointly by GPs in Norway and GPs and sociologists in France. Initially, British, Belgian and Italian GPs participated in discussions, and a preliminary questionnaire was tested in five countries [6]. The current team concentrated on two countries: France and Norway. The questionnaire was originally in French and was translated into Norwegian by one of the authors (KH). An intra-observer test-retest reliability study [7] was performed in Norway where GPs completed the same questionnaire twice with one month's interval for the same 15 patients. The re-test was not announced when the questionnaire was first sent out. Total agreement in 660 answers was 88.2% (95% CI 85.7-90.6%). This was considered satisfactory.

In the present study, the data were drawn from the cancer patients' routine medical records. Their GPs answered the 40 closed-ended questions on the patients' care (prevention, diagnostic process, choice of medical team and initial treatment, monitoring of the disease and its side-effects, end-of-life if relevant) and on the doctor-patient relationship (length of the relationship, psychological and social support, contact with the family). The patient inclusion protocol consisted of two conditions:

- having or having had cancer, irrespective of the location of the disease, or of its stage or whether the patient was still alive;

- having consulted or seen the doctor at least once in the year preceding the survey.

In France, the GPs were asked to select the five first patients on the doctor's list of consultations, who met the inclusion criteria; while in Norway the GPs were asked to select one or two patients they remembered, given the inclusion criteria.

### Data collection

In France the postal questionnaires were sent out in January of 2005 to a 10% sample of GPs practicing in mainland France (n = 5056). This was a random sample identified by the national health fund (CNAMTS). In Norway the postal questionnaires were sent out in October 2005 to a 10% random sample of South Norwegian GPs and to all the GPs in the sparsely populated North Norway (n = 801). This sample was supplied by the Norwegian Medical Association (NMA). In France, 1679 eligible patient questionnaires were filled in by 348 GPs (7% of the GP sample), on average 4.8 questionnaires per respondent. In Norway, 386 eligible patient questionnaires were filled in by 292 GPs (39% of the GP sample) with an average of 1.3 questionnaires per respondent.

The data were plotted and analysed using SPSS. Because of the differences in the instructions given to the doctors in the two countries, and also because of low response rates, we are not presenting statistical comparisons between the two countries. Chi square tests and t-tests are used for within-country analyses.

### Ethical approval

The survey protocol was accepted by the French Data Protection Authority (CNIL). No patients were contacted, and personal data recorded were limited to sex, year of birth and type of cancer. Clearance by Ethical Research Committees was therefore not deemed necessary.

## Results

### The cancer patients

Age and sex of the patients are presented in table 1. Mean age at diagnosis was 61 years in France and 60 years in Norway; males were older than females in both countries. Health status of the patients at the time of the most recent consultation is shown in table 2. Three types of cancer - breast, colorectal, and male genital organs - predominated, with a majority of breast cancers among French patients (27% France, 17% Norway) and colorectal cancers for Norwegian patients (13% France, 21% Norway). 182 (11%) French and 12 Norwegian patients (3%) had their second cancer.

**Table 1:** Patient sex and age in years

	**France (N = 1697)**				**Norway (N = 386)**			
	**Females**	**Males**	**All**	**P**^1^	**Females**	**Males**	**All**	**P**^1^
Mean age at diagnosis	58	63	61	<0.001	57	63	60	<0.001
Median age at diagnosis	58	65	62		58	65	61	
Mean age at last follow-up	63	66	65	<0.001	60	66	63	<0.001
Median age at last follow-up	64	68	67		61	68	63	

1. Within-country difference between females and males, t-test.

**Table 2:** Clinical status of the patients^1^

	**France**		**Norway**	
**Clinical status**	**N**	**%**	**N**	**%**
Well, no current cancer treatment	686	41.0	105	27.4
Undergoing treatment for cancer	638	38.1	171	44.6
In terminal phase	104	6.2	29	7.6
Dead	247	14.7	78	20.4
Sum	1675	100	383	100

^1^Data missing for 22 French patients and 3 Norwegian patients

### The GP-patient relationship

80% of French patients and 73% of Norwegian patients consulted the GP before their cancer was diagnosed. In 9% of French cases and 18% of Norwegian cases, the cancer diagnosis and the first meeting with this GP took place in the same year.

### Prevention at the GP's surgery

GPs commonly discuss risks relating to life-style, environment or family medical history with their patients. For the cancer patients in our study, such preventive information before diagnosis had been given to 42% of Norwegian patients and 30% of French patients. Previous preventive action in the form of screening or case finding without a presumption of cancer on clinical grounds had been carried out for about half of the patients in both countries.

### Establishing a diagnosis (table 3)

**Table 3:** GP's role in diagnosis and therapy

	**France**		**Norway**	
Diagnostic process	N	%	N	%
Diagnosis after clinical suspicion	994	59	282	73
Chance Discovery	270	16	54	14
Diagnosis after screening in asymptomatic patient	313	19	32	9
Implication of GP in the diagnostic procedure	1310	78	319	83
Personal GP took diagnostic initiative	1223	73	257	67
Another GP took diagnostic initiative	87	5	62	16
Patient feared having cancer	455	27	33	9
Therapy				
Organisation of health care decided treatment team	259	15	309	80
Treatment team reputation contributed to choice	820	49	54	14
Patient or family contributed to choice	489	29	47	12
GP implicated in choice of cancer treatment	223	13	15	4
GP implicated in dealing with side effects of treatment	910	54	180	47
GP implicated in administering chemotherapy	68	4	34	9

GP = General Practitioner

In both countries, GPs reported to a large extent being involved in the diagnosis. The GPs considered themselves to be at the origin of the process of discovering the cancer for 78% of the patients in France and for 83% in Norway. This varied for different forms of cancer (P < 0.001 for France, P = 0.003 for Norway), and was most marked for colorectal cancer and male genital cancer in both countries. For breast cancer, only about six of every ten cases had been diagnosed by GPs in both countries. In France, more males than females were diagnosed by the GP (P < 0.001). The patients diagnosed by GPs were older, 62 vs 57 years (P < 0.001) in France, and 61 vs 57 years in Norway (P = 0.03).

In most cases the cancer was discovered after a clinical or complementary procedure, based on a suspicion of cancer (59% France, 73% Norway) and usually prescribed by the GP him/herself. The rate of chance discovery was similar, 16% in France and 14% in Norway. Diagnosis after screening of an asymptomatic patient was 19% among French patients and 9% among Norwegian patients. The screening difference is mainly due to a much higher percentage of screened cases of male genital cancer, mainly of the prostate. Male genital cancer constituted 14% of all cases in both countries. In France 45% of these patients had been diagnosed after screening while in Norway this was the case for 14%.

### GPs' role in therapy (table 3)

Choice of the medical team differs structurally because the existence of pre-established treatment trajectories in Norway determines the choice for a majority of cancer patients. In France, a more or less "compulsory" team was imposed for only 15% of patients. The choice can be based on the GP's personal relations and/or the team's reputation (49% France, 14% Norway), while taking into account the patient's and his/her family's wishes, if they are expressed (29% France, 12% Norway).

### Involvement in follow-up after cancer treatment (table 4)

**Table 4:** Participation in follow-up by the general practitioner during or after primary cancer treatment

	**France** **N = 1679**		**Norway** **N = 386**	
**Procedure**	**N**	**%**	**N**	**%**
GP implicated in follow-up 1^st ^year after primary treatment	1277	76	252	65
GP implicated in follow-up after first year	1179	78^1^	215	73^1^
Important patient-doctor talk	1309	78	327	85
Social-administrative help (sick leave, home based care...)	963	57	259	67
Co-ordination of home care	369	22	67	17
Treatment of non-cancer disease	1203	72	247	64
Non-cancer treatment led to contact with cancer therapist	281	17	45	12

^1 ^Of 1519 French patients and 295 Norwegian patients where this was relevant one year after primary treatment

During the first year of the follow-up, the GP was involved in the care for about three quarters of French patients and for about two thirds of Norwegian patients, mainly in collaboration with the hospital team and with other professionals. The GPs continued to be involved in follow-up after the first year for the great majority of their patients. In both countries GPs gave considerable psychological support to their patients, including conversations, assistance with administrative procedures and, to a lesser extent, coordination of home care. A majority of the patients continued consulting a GP for other ailments than their cancer.

### GP involvement for patients with progressive disease (table 5)

**Table 5:** GP participation in progressive disease, treatment with opiates, place where the person received terminal care and died, and contact with the family after the patient's death

	**France**		**Norway**	
	**N**	**%**	**N**	**%**
GP participated in care after clinical aggravation or relapse	392	70^1^	138	66^1^
Opiates prescribed for home use ^2^	399	24	122	32
By GP alone	214	54	42	35
By GP + hospital/pain team	100	25	40	33
By hospital/pain team	85	21	40	33
Location of terminal care^3^:				
Home or home institution	220	70	86	80
Special unit for palliative care	32	10	12	11
Hospital	144	45	49	46
Hereof only in hospital	99	31	20	19
Dead:	247	15	78	20
Hereof at home	100	41	21	27
Contact with family after death	217	88	55	71

^1 ^Of 561 French patients and 208 Norwegian patients with progressive disease or relapse.

^2 ^Data missing for 3 Norwegian patients

^3 ^Counting patients in terminal phase or dead

For 561 French and 208 Norwegian patients, the GPs reported that the cancer had progressed or relapsed. GPs participated in the care in the majority of these cases. One fourth of all French patients and one third of all Norwegian patients received morphine prescribed by their GP; the difference can be explained by the greater proportion of dead and terminal patients in Norway. No differences were found within either country for different diagnostic groups or for sex or age. In many cases, collaboration with hospital colleagues had been established in these situations. In both countries approximately half of the patients spent their entire terminal phase either at home or in a retirement facility [8].

## Discussion

### Questions of method

There are no validated questionnaires assessing GPs' role in cancer care. To collect data about cancer patients, we therefore gathered a panel of European GPs to develop the questionnaire based on information about individual patients available in medical records, and which could be filled in by the GP. A test-retest study in Norway found the reliability satisfactory. Most questions seem to have been readily understood in the two countries. Recall bias has been reduced by the use of medical records when the information was reported, and the internal validity seems to be satisfactory in that the answers give a rather comprehensive picture of what French and Norwegian GPs do in cancer care. For consideration of further validity the questionnaire would have to be tested in other countries.

The strength of our approach is that the GPs were reporting what happened for specific patients rather than expressing their opinions about what happens in general. The time required for GPs to fill in five patient questionnaires probably explains the low rate of response by French GPs. The better response rate in Norway may reflect that only one or two cancer cases were asked for, and that GPs were not asked to search records in order to find patients. However, it probably contributed to inequalities in the two samples of cancer patients and thus to some of the observed patient differences. Norwegian GPs selected, to a greater extent than their French counterparts, patients who were more present in their minds. This could be patients being actively treated for cancer, those in terminal phase or those who had recently passed away, rather than older patients who had recovered. Going back in the list of consultations, as the French protocol required, may have minimized the effect of memory. Also, including more files per GP may have increased the probability of including patients who were in remission or cured. The response rate in both countries is far too low to be representative for either all GPs or for all cancer patients. This severely limits the validity of country comparisons and is why we have avoided statistical between-countries comparisons. However, it does not invalidate findings of a high level of cancer care activities in both countries, and several of our findings are consistent with English data [9]. For Norwegian doctors, the sex distribution was no different for responders and non-responders. For non-responders, we do not have further data. In France female doctors were slightly overrepresented among responders, while there was no difference in mean age.

### GPs' participation in diagnosis

A previous study from England has suggested that the role of the GP in cancer care is important [9]. The variation in the GP's diagnostic role associated with cancer type was found in this study as well. The essential role of GPs in the discovery of cancer is confirmed in our study. Norwegian GPs generally have access in their own group practices to a greater repertoire of supplementary tests than French GPs, who must refer patients for blood tests. This may have had some impact on detecting cancer on clinical grounds. Nevertheless, GPs' involvement in diagnostics was shown to be common in both countries. Differences in how our national samples were collected tend to introduce bias in any comparisons. However, we think that the considerable difference in the proportion of screened cases of prostate cancer suggests that French GPs are more prone to use PSA screening, a hotly debated issue with no clear recommendations in France and a negative recommendation in Norway. This difference may explain that the GP's diagnostic role was more important for males in France, but not in Norway.

Good quality GP work during the diagnostic process is important even if this is less relevant for the substantial minority of cases diagnosed by screening or presenting as emergencies. The clinical challenge for the GP is considerable, emphasized by a British study which for six cancers showed longer diagnostic delay for patients having seen their GP prior to diagnosis than for those who did not [10]. Possibilities for improvement must be considered in the context of the low predictive value of suspected symptoms and the challenges attached to the use of watchful waiting and appropriate thresholds for referral. The low specificity of possible cancer symptoms is well known and is a major challenge for GPs and other doctors consulted prior to diagnosis [11]. GPs know that age is a major risk factor for cancer, and our study confirms that attention to this is justified in that older patients more than younger patients consulted a GP before diagnosis. Fear of cancer in consulting patients should be taken seriously, but is generally of little value in making a cancer diagnosis [12].

### GPs' participation in treatment

After the initial treatment, largely defined and carried out in a hospital, psychosocial follow-up and treatment of non-cancer ailments are important tasks for the GP. Treating side-effects is common. Our study did not ask about follow-up for specific cancers. Others have shown that clinical follow-up of breast cancer by the GP does not increase delay in diagnosing relapse [13]. GPs can also play an important role in the follow-up of colorectal cancer [14].

In the terminal phase, GP involvement is desirable and possible [15]. French GPs are more often involved in treating patients at home than are Norwegian GPs, although their role is important in both countries. The system in Norway seems to allow for a return to hospital or to a specialized service more easily than in France.

## Conclusion

French and Norwegian GPs participate actively in care for cancer patients. Cancer diagnostics is a challenge for the GP when a patient fears or the GP suspects cancer. This is consistent in both France and Norway, and it calls for more research about how GPs can improve their early diagnostic work. The organisation of care differs, and pre-established treatment trajectories or a predefined role as a personal doctor may encourage other cancer care tasks for the GP than a system that emphasizes the maintenance of the patient's and doctor's freedom of choice. Further national studies could explore possible new tasks for the GP in cancer care. Most patients in both countries return to their GP some time after finishing treatment, either for non-cancer ailments or for progression of cancer symptoms. In the latter case, GPs and hospital doctors need to cooperate and this should be reflected in their medical training.

## Abbreviations used

GP: General practitioner.

## Competing interests

We declare that we have no conflicts of interest.

This work was supported by Fondation de France, Caisse Nationale de l'Assurance Maladie des Travailleurs Salariés (CNAMTS), Institut National du Cancer (INCA) and by the Norwegian Medical Association through a general practice research grant

## Authors' contributions

MB, AL and KH contributed to the creation of the questionnaire. KH conducted the pilot intra-observer study. KH and TT collected the Norwegian data. JB, AL and MB collected the French data. LD and KH performed statistical analyses. LD, MB and KH wrote the first draft of the article. All authors read and approved the final manuscript.

## Supplementary Material

Additional file 1 **Patient questionnaire, English version**. Questionnaire with data about each cancer patient, completed by the patient's general practitioner Click here for file
